# Supplementary material for: Pyruvate Dehydrogenase Kinase 1 inhibition mediated oxidative phosphorylation enhancement in cartilage promotes osteoarthritis progression
Source: BMC Musculoskelet Disord. 2023 Jul 20;24:597. doi: 10.1186/s12891-023-06585-6 (PMC10357736; doi:10.1186/s12891-023-06585-6)
Supplement: Supplementary file 1 — Additional file 1. [file 12891_2023_6585_MOESM1_ESM.zip › MF.Gsea.1653627316242/GOMF_NUCLEOSIDE_TRIPHOSPHATASE_REGULATOR_ACTIVITY.html]

Details for gene set GOMF\_NUCLEOSIDE\_TRIPHOSPHATASE\_REGULATOR\_ACTIVITY[GSEA]

|  || Dataset | 55457\_collapsed\_to\_symbols.cls.cls #OA\_versus\_normal.cls.cls #OA\_versus\_normal\_repos |
| Phenotype | cls.cls#OA\_versus\_normal\_repos |
| Upregulated in class | normal |
| GeneSet | GOMF\_NUCLEOSIDE\_TRIPHOSPHATASE\_REGULATOR\_ACTIVITY |
| Enrichment Score (ES) | -0.2719412 |
| Normalized Enrichment Score (NES) | -1.3832258 |
| Nominal p-value | 0.005249344 |
| FDR q-value | 0.2834267 |
| FWER p-Value | 1.0 |
Table: GSEA Results Summary

  

Fig 1: Enrichment plot: GOMF\_NUCLEOSIDE\_TRIPHOSPHATASE\_REGULATOR\_ACTIVITY      
 Profile of the Running ES Score & Positions of GeneSet Members on the Rank Ordered List

  

| SYMBOL | TITLE | RANK IN GENE LIST | RANK METRIC SCORE | RUNNING ES | CORE ENRICHMENT || 1 | ADGRB3 | adhesion G protein-coupled receptor B3 [Source:HGNC Symbol;Acc:HGNC:945] | 131 | 0.874 | -0.0026 | No |
| 2 | LAMTOR5 | "late endosomal/lysosomal adaptor, MAPK and MTOR activator 5 [Source:HGNC Symbol;Acc:HGNC:17955]" | 187 | 0.829 | 0.0005 | No |
| 3 | ARL2BP | ADP ribosylation factor like GTPase 2 binding protein [Source:HGNC Symbol;Acc:HGNC:17146] | 208 | 0.813 | 0.0062 | No |
| 4 | RALBP1 | ralA binding protein 1 [Source:HGNC Symbol;Acc:HGNC:9841] | 217 | 0.808 | 0.0128 | No |
| 5 | TBC1D4 | TBC1 domain family member 4 [Source:HGNC Symbol;Acc:HGNC:19165] | 320 | 0.737 | 0.0113 | No |
| 6 | ELMO1 | engulfment and cell motility 1 [Source:HGNC Symbol;Acc:HGNC:16286] | 370 | 0.718 | 0.0139 | No |
| 7 | NPRL2 | "NPR2 like, GATOR1 complex subunit [Source:HGNC Symbol;Acc:HGNC:24969]" | 429 | 0.695 | 0.0155 | No |
| 8 | RGS4 | regulator of G protein signaling 4 [Source:HGNC Symbol;Acc:HGNC:10000] | 449 | 0.683 | 0.0201 | No |
| 9 | DENND1B | DENN domain containing 1B [Source:HGNC Symbol;Acc:HGNC:28404] | 450 | 0.683 | 0.0263 | No |
| 10 | DOCK2 | dedicator of cytokinesis 2 [Source:HGNC Symbol;Acc:HGNC:2988] | 520 | 0.652 | 0.0267 | No |
| 11 | TBC1D19 | TBC1 domain family member 19 [Source:HGNC Symbol;Acc:HGNC:25624] | 528 | 0.648 | 0.0319 | No |
| 12 | HTR2B | 5-hydroxytryptamine receptor 2B [Source:HGNC Symbol;Acc:HGNC:5294] | 562 | 0.636 | 0.0350 | No |
| 13 | VAV1 | vav guanine nucleotide exchange factor 1 [Source:HGNC Symbol;Acc:HGNC:12657] | 601 | 0.622 | 0.0376 | No |
| 14 | EIF2B2 | eukaryotic translation initiation factor 2B subunit beta [Source:HGNC Symbol;Acc:HGNC:3258] | 627 | 0.612 | 0.0411 | No |
| 15 | ITGB1BP1 | integrin subunit beta 1 binding protein 1 [Source:HGNC Symbol;Acc:HGNC:23927] | 631 | 0.611 | 0.0464 | No |
| 16 | RGS10 | regulator of G protein signaling 10 [Source:HGNC Symbol;Acc:HGNC:9992] | 635 | 0.610 | 0.0516 | No |
| 17 | ARHGEF18 | Rho/Rac guanine nucleotide exchange factor 18 [Source:HGNC Symbol;Acc:HGNC:17090] | 645 | 0.606 | 0.0563 | No |
| 18 | RANGRF | RAN guanine nucleotide release factor [Source:HGNC Symbol;Acc:HGNC:17679] | 660 | 0.603 | 0.0606 | No |
| 19 | PDE6D | phosphodiesterase 6D [Source:HGNC Symbol;Acc:HGNC:8788] | 718 | 0.589 | 0.0614 | No |
| 20 | SERGEF | secretion regulating guanine nucleotide exchange factor [Source:HGNC Symbol;Acc:HGNC:17499] | 864 | 0.552 | 0.0548 | No |
| 21 | FGD6 | "FYVE, RhoGEF and PH domain containing 6 [Source:HGNC Symbol;Acc:HGNC:21740]" | 930 | 0.532 | 0.0544 | No |
| 22 | EPS8L2 | EPS8 like 2 [Source:HGNC Symbol;Acc:HGNC:21296] | 1134 | 0.489 | 0.0426 | No |
| 23 | RIC8A | RIC8 guanine nucleotide exchange factor A [Source:HGNC Symbol;Acc:HGNC:29550] | 1224 | 0.471 | 0.0398 | No |
| 24 | SMAP1 | small ArfGAP 1 [Source:HGNC Symbol;Acc:HGNC:19651] | 1243 | 0.468 | 0.0426 | No |
| 25 | LAMTOR2 | "late endosomal/lysosomal adaptor, MAPK and MTOR activator 2 [Source:HGNC Symbol;Acc:HGNC:29796]" | 1283 | 0.461 | 0.0436 | No |
| 26 | RGS20 | regulator of G protein signaling 20 [Source:HGNC Symbol;Acc:HGNC:14600] | 1345 | 0.451 | 0.0428 | No |
| 27 | CCDC88C | coiled-coil domain containing 88C [Source:HGNC Symbol;Acc:HGNC:19967] | 1455 | 0.435 | 0.0380 | No |
| 28 | ARHGEF9 | Cdc42 guanine nucleotide exchange factor 9 [Source:HGNC Symbol;Acc:HGNC:14561] | 1469 | 0.433 | 0.0409 | No |
| 29 | ASAP3 | "ArfGAP with SH3 domain, ankyrin repeat and PH domain 3 [Source:HGNC Symbol;Acc:HGNC:14987]" | 1480 | 0.432 | 0.0440 | No |
| 30 | SIPA1 | signal-induced proliferation-associated 1 [Source:HGNC Symbol;Acc:HGNC:10885] | 1488 | 0.431 | 0.0473 | No |
| 31 | PLCE1 | phospholipase C epsilon 1 [Source:HGNC Symbol;Acc:HGNC:17175] | 1505 | 0.428 | 0.0498 | No |
| 32 | RCC1L | RCC1 like [Source:HGNC Symbol;Acc:HGNC:14948] | 1559 | 0.419 | 0.0494 | No |
| 33 | DENND2D | DENN domain containing 2D [Source:HGNC Symbol;Acc:HGNC:26192] | 1656 | 0.406 | 0.0454 | No |
| 34 | ADAP1 | ArfGAP with dual PH domains 1 [Source:HGNC Symbol;Acc:HGNC:16486] | 1706 | 0.398 | 0.0451 | No |
| 35 | TBC1D22A | TBC1 domain family member 22A [Source:HGNC Symbol;Acc:HGNC:1309] | 1718 | 0.395 | 0.0477 | No |
| 36 | ALDH1A1 | aldehyde dehydrogenase 1 family member A1 [Source:HGNC Symbol;Acc:HGNC:402] | 1737 | 0.393 | 0.0498 | No |
| 37 | RAB3GAP1 | RAB3 GTPase activating protein catalytic subunit 1 [Source:HGNC Symbol;Acc:HGNC:17063] | 1766 | 0.389 | 0.0511 | No |
| 38 | ARHGAP25 | Rho GTPase activating protein 25 [Source:HGNC Symbol;Acc:HGNC:28951] | 1900 | 0.373 | 0.0439 | No |
| 39 | TBC1D5 | TBC1 domain family member 5 [Source:HGNC Symbol;Acc:HGNC:19166] | 1956 | 0.367 | 0.0428 | No |
| 40 | USP6NL | USP6 N-terminal like [Source:HGNC Symbol;Acc:HGNC:16858] | 1999 | 0.361 | 0.0427 | No |
| 41 | ARHGAP24 | Rho GTPase activating protein 24 [Source:HGNC Symbol;Acc:HGNC:25361] | 2040 | 0.357 | 0.0427 | No |
| 42 | PTGIR | prostaglandin I2 receptor [Source:HGNC Symbol;Acc:HGNC:9602] | 2055 | 0.356 | 0.0448 | No |
| 43 | ARRB1 | arrestin beta 1 [Source:HGNC Symbol;Acc:HGNC:711] | 2116 | 0.348 | 0.0431 | No |
| 44 | WDR41 | WD repeat domain 41 [Source:HGNC Symbol;Acc:HGNC:25601] | 2134 | 0.346 | 0.0449 | No |
| 45 | DENND1A | DENN domain containing 1A [Source:HGNC Symbol;Acc:HGNC:29324] | 2189 | 0.340 | 0.0436 | No |
| 46 | RABGAP1L | RAB GTPase activating protein 1 like [Source:HGNC Symbol;Acc:HGNC:24663] | 2334 | 0.322 | 0.0351 | No |
| 47 | NUCB2 | nucleobindin 2 [Source:HGNC Symbol;Acc:HGNC:8044] | 2365 | 0.319 | 0.0355 | No |
| 48 | TBC1D2B | TBC1 domain family member 2B [Source:HGNC Symbol;Acc:HGNC:29183] | 2375 | 0.317 | 0.0377 | No |
| 49 | EGF | epidermal growth factor [Source:HGNC Symbol;Acc:HGNC:3229] | 2399 | 0.315 | 0.0387 | No |
| 50 | ARHGAP15 | Rho GTPase activating protein 15 [Source:HGNC Symbol;Acc:HGNC:21030] | 2420 | 0.312 | 0.0399 | No |
| 51 | RALGAPB | Ral GTPase activating protein non-catalytic subunit beta [Source:HGNC Symbol;Acc:HGNC:29221] | 2426 | 0.311 | 0.0423 | No |
| 52 | RABIF | RAB interacting factor [Source:HGNC Symbol;Acc:HGNC:9797] | 2488 | 0.304 | 0.0402 | No |
| 53 | RASA1 | RAS p21 protein activator 1 [Source:HGNC Symbol;Acc:HGNC:9871] | 2506 | 0.302 | 0.0415 | No |
| 54 | RAPGEFL1 | Rap guanine nucleotide exchange factor like 1 [Source:HGNC Symbol;Acc:HGNC:17428] | 2514 | 0.302 | 0.0437 | No |
| 55 | TBC1D16 | TBC1 domain family member 16 [Source:HGNC Symbol;Acc:HGNC:28356] | 2536 | 0.299 | 0.0447 | No |
| 56 | ARFGAP2 | ADP ribosylation factor GTPase activating protein 2 [Source:HGNC Symbol;Acc:HGNC:13504] | 2548 | 0.297 | 0.0465 | No |
| 57 | CCZ1 | "CCZ1 homolog, vacuolar protein trafficking and biogenesis associated [Source:HGNC Symbol;Acc:HGNC:21691]" | 2558 | 0.296 | 0.0484 | No |
| 58 | NCKAP1L | NCK associated protein 1 like [Source:HGNC Symbol;Acc:HGNC:4862] | 2560 | 0.296 | 0.0510 | No |
| 59 | DOCK10 | dedicator of cytokinesis 10 [Source:HGNC Symbol;Acc:HGNC:23479] | 2605 | 0.290 | 0.0501 | No |
| 60 | LAMTOR3 | "late endosomal/lysosomal adaptor, MAPK and MTOR activator 3 [Source:HGNC Symbol;Acc:HGNC:15606]" | 2674 | 0.283 | 0.0472 | No |
| 61 | RHOH | ras homolog family member H [Source:HGNC Symbol;Acc:HGNC:686] | 2680 | 0.282 | 0.0494 | No |
| 62 | HERC1 | HECT and RLD domain containing E3 ubiquitin protein ligase family member 1 [Source:HGNC Symbol;Acc:HGNC:4867] | 2684 | 0.281 | 0.0517 | No |
| 63 | STARD8 | StAR related lipid transfer domain containing 8 [Source:HGNC Symbol;Acc:HGNC:19161] | 2785 | 0.271 | 0.0461 | No |
| 64 | IQSEC1 | IQ motif and Sec7 domain ArfGEF 1 [Source:HGNC Symbol;Acc:HGNC:29112] | 2814 | 0.269 | 0.0463 | No |
| 65 | GMIP | GEM interacting protein [Source:HGNC Symbol;Acc:HGNC:24852] | 2835 | 0.267 | 0.0471 | No |
| 66 | RGS7 | regulator of G protein signaling 7 [Source:HGNC Symbol;Acc:HGNC:10003] | 2842 | 0.266 | 0.0490 | No |
| 67 | ARHGEF5 | Rho guanine nucleotide exchange factor 5 [Source:HGNC Symbol;Acc:HGNC:13209] | 2938 | 0.257 | 0.0438 | No |
| 68 | DENND2B | DENN domain containing 2B [Source:HGNC Symbol;Acc:HGNC:11350] | 2957 | 0.254 | 0.0446 | No |
| 69 | FAM13A | family with sequence similarity 13 member A [Source:HGNC Symbol;Acc:HGNC:19367] | 3048 | 0.245 | 0.0397 | No |
| 70 | MCF2L2 | MCF.2 cell line derived transforming sequence-like 2 [Source:HGNC Symbol;Acc:HGNC:30319] | 3133 | 0.237 | 0.0351 | No |
| 71 | RANBP1 | RAN binding protein 1 [Source:HGNC Symbol;Acc:HGNC:9847] | 3214 | 0.229 | 0.0308 | No |
| 72 | RGS3 | regulator of G protein signaling 3 [Source:HGNC Symbol;Acc:HGNC:9999] | 3247 | 0.226 | 0.0303 | No |
| 73 | TBC1D9 | TBC1 domain family member 9 [Source:HGNC Symbol;Acc:HGNC:21710] | 3287 | 0.222 | 0.0291 | No |
| 74 | ARHGEF4 | Rho guanine nucleotide exchange factor 4 [Source:HGNC Symbol;Acc:HGNC:684] | 3307 | 0.220 | 0.0296 | No |
| 75 | DENND1C | DENN domain containing 1C [Source:HGNC Symbol;Acc:HGNC:26225] | 3358 | 0.216 | 0.0276 | No |
| 76 | EIF2B3 | eukaryotic translation initiation factor 2B subunit gamma [Source:HGNC Symbol;Acc:HGNC:3259] | 3461 | 0.206 | 0.0213 | No |
| 77 | CYTH2 | cytohesin 2 [Source:HGNC Symbol;Acc:HGNC:9502] | 3486 | 0.204 | 0.0212 | No |
| 78 | RUNDC3A | RUN domain containing 3A [Source:HGNC Symbol;Acc:HGNC:16984] | 3501 | 0.202 | 0.0219 | No |
| 79 | ARHGEF10 | Rho guanine nucleotide exchange factor 10 [Source:HGNC Symbol;Acc:HGNC:14103] | 3540 | 0.199 | 0.0207 | No |
| 80 | SIRPA | signal regulatory protein alpha [Source:HGNC Symbol;Acc:HGNC:9662] | 3599 | 0.193 | 0.0178 | No |
| 81 | NET1 | neuroepithelial cell transforming 1 [Source:HGNC Symbol;Acc:HGNC:14592] | 3607 | 0.193 | 0.0190 | No |
| 82 | PLEKHG6 | pleckstrin homology and RhoGEF domain containing G6 [Source:HGNC Symbol;Acc:HGNC:25562] | 3662 | 0.189 | 0.0164 | No |
| 83 | PSD3 | pleckstrin and Sec7 domain containing 3 [Source:HGNC Symbol;Acc:HGNC:19093] | 3672 | 0.188 | 0.0173 | No |
| 84 | NRP1 | neuropilin 1 [Source:HGNC Symbol;Acc:HGNC:8004] | 3704 | 0.185 | 0.0165 | No |
| 85 | PLCB1 | phospholipase C beta 1 [Source:HGNC Symbol;Acc:HGNC:15917] | 3724 | 0.183 | 0.0167 | No |
| 86 | SEC23B | "SEC23 homolog B, COPII coat complex component [Source:HGNC Symbol;Acc:HGNC:10702]" | 3838 | 0.172 | 0.0092 | No |
| 87 | EIF2B4 | eukaryotic translation initiation factor 2B subunit delta [Source:HGNC Symbol;Acc:HGNC:3260] | 3878 | 0.167 | 0.0076 | No |
| 88 | TIAM1 | TIAM Rac1 associated GEF 1 [Source:HGNC Symbol;Acc:HGNC:11805] | 3967 | 0.159 | 0.0020 | No |
| 89 | MCF2 | MCF.2 cell line derived transforming sequence [Source:HGNC Symbol;Acc:HGNC:6940] | 3991 | 0.157 | 0.0016 | No |
| 90 | RCBTB2 | RCC1 and BTB domain containing protein 2 [Source:HGNC Symbol;Acc:HGNC:1914] | 4014 | 0.155 | 0.0012 | No |
| 91 | SEC23A | "SEC23 homolog A, COPII coat complex component [Source:HGNC Symbol;Acc:HGNC:10701]" | 4017 | 0.154 | 0.0025 | No |
| 92 | TBC1D8B | TBC1 domain family member 8B [Source:HGNC Symbol;Acc:HGNC:24715] | 4040 | 0.152 | 0.0021 | No |
| 93 | ARHGDIG | Rho GDP dissociation inhibitor gamma [Source:HGNC Symbol;Acc:HGNC:680] | 4063 | 0.150 | 0.0017 | No |
| 94 | VAV3 | vav guanine nucleotide exchange factor 3 [Source:HGNC Symbol;Acc:HGNC:12659] | 4082 | 0.148 | 0.0016 | No |
| 95 | ARHGDIB | Rho GDP dissociation inhibitor beta [Source:HGNC Symbol;Acc:HGNC:679] | 4085 | 0.147 | 0.0027 | No |
| 96 | DIS3 | "DIS3 homolog, exosome endoribonuclease and 3'-5' exoribonuclease [Source:HGNC Symbol;Acc:HGNC:20604]" | 4187 | 0.139 | -0.0041 | No |
| 97 | RABGEF1 | RAB guanine nucleotide exchange factor 1 [Source:HGNC Symbol;Acc:HGNC:17676] | 4192 | 0.139 | -0.0031 | No |
| 98 | TBC1D1 | TBC1 domain family member 1 [Source:HGNC Symbol;Acc:HGNC:11578] | 4215 | 0.136 | -0.0037 | No |
| 99 | RAP1GDS1 | Rap1 GTPase-GDP dissociation stimulator 1 [Source:HGNC Symbol;Acc:HGNC:9859] | 4234 | 0.134 | -0.0039 | No |
| 100 | RGS11 | regulator of G protein signaling 11 [Source:HGNC Symbol;Acc:HGNC:9993] | 4304 | 0.128 | -0.0082 | No |
| 101 | ARHGAP11A | Rho GTPase activating protein 11A [Source:HGNC Symbol;Acc:HGNC:15783] | 4326 | 0.126 | -0.0088 | No |
| 102 | DOCK3 | dedicator of cytokinesis 3 [Source:HGNC Symbol;Acc:HGNC:2989] | 4381 | 0.122 | -0.0120 | No |
| 103 | ARHGAP45 | Rho GTPase activating protein 45 [Source:HGNC Symbol;Acc:HGNC:17102] | 4402 | 0.121 | -0.0125 | No |
| 104 | THG1L | tRNA-histidine guanylyltransferase 1 like [Source:HGNC Symbol;Acc:HGNC:26053] | 4417 | 0.120 | -0.0125 | No |
| 105 | RALGPS1 | Ral GEF with PH domain and SH3 binding motif 1 [Source:HGNC Symbol;Acc:HGNC:16851] | 4469 | 0.115 | -0.0156 | No |
| 106 | DEPDC1 | DEP domain containing 1 [Source:HGNC Symbol;Acc:HGNC:22949] | 4482 | 0.114 | -0.0155 | No |
| 107 | LLGL2 | LLGL scribble cell polarity complex component 2 [Source:HGNC Symbol;Acc:HGNC:6629] | 4604 | 0.104 | -0.0242 | No |
| 108 | DOCK4 | dedicator of cytokinesis 4 [Source:HGNC Symbol;Acc:HGNC:19192] | 4607 | 0.104 | -0.0234 | No |
| 109 | ALS2CL | ALS2 C-terminal like [Source:HGNC Symbol;Acc:HGNC:20605] | 4668 | 0.099 | -0.0273 | No |
| 110 | WNT11 | Wnt family member 11 [Source:HGNC Symbol;Acc:HGNC:12776] | 4688 | 0.097 | -0.0280 | No |
| 111 | ARAP1 | "ArfGAP with RhoGAP domain, ankyrin repeat and PH domain 1 [Source:HGNC Symbol;Acc:HGNC:16925]" | 4730 | 0.093 | -0.0304 | No |
| 112 | FARP1 | "FERM, ARH/RhoGEF and pleckstrin domain protein 1 [Source:HGNC Symbol;Acc:HGNC:3591]" | 4744 | 0.092 | -0.0306 | No |
| 113 | PSD4 | pleckstrin and Sec7 domain containing 4 [Source:HGNC Symbol;Acc:HGNC:19096] | 4749 | 0.091 | -0.0301 | No |
| 114 | ARHGEF3 | Rho guanine nucleotide exchange factor 3 [Source:HGNC Symbol;Acc:HGNC:683] | 4845 | 0.084 | -0.0369 | No |
| 115 | TBC1D12 | TBC1 domain family member 12 [Source:HGNC Symbol;Acc:HGNC:29082] | 4931 | 0.078 | -0.0430 | No |
| 116 | THY1 | Thy-1 cell surface antigen [Source:HGNC Symbol;Acc:HGNC:11801] | 4985 | 0.074 | -0.0466 | No |
| 117 | DOCK1 | dedicator of cytokinesis 1 [Source:HGNC Symbol;Acc:HGNC:2987] | 4998 | 0.073 | -0.0469 | No |
| 118 | RAB3IL1 | RAB3A interacting protein like 1 [Source:HGNC Symbol;Acc:HGNC:9780] | 5081 | 0.066 | -0.0528 | No |
| 119 | ACAP1 | "ArfGAP with coiled-coil, ankyrin repeat and PH domains 1 [Source:HGNC Symbol;Acc:HGNC:16467]" | 5210 | 0.056 | -0.0625 | No |
| 120 | OBSCN | "obscurin, cytoskeletal calmodulin and titin-interacting RhoGEF [Source:HGNC Symbol;Acc:HGNC:15719]" | 5230 | 0.054 | -0.0635 | No |
| 121 | ARHGEF40 | Rho guanine nucleotide exchange factor 40 [Source:HGNC Symbol;Acc:HGNC:25516] | 5357 | 0.043 | -0.0732 | No |
| 122 | SH3BP4 | SH3 domain binding protein 4 [Source:HGNC Symbol;Acc:HGNC:10826] | 5470 | 0.035 | -0.0818 | No |
| 123 | ADAP2 | ArfGAP with dual PH domains 2 [Source:HGNC Symbol;Acc:HGNC:16487] | 5535 | 0.030 | -0.0866 | No |
| 124 | FAM13B | family with sequence similarity 13 member B [Source:HGNC Symbol;Acc:HGNC:1335] | 5589 | 0.025 | -0.0906 | No |
| 125 | CCDC88A | coiled-coil domain containing 88A [Source:HGNC Symbol;Acc:HGNC:25523] | 5641 | 0.020 | -0.0945 | No |
| 126 | NUCB1 | nucleobindin 1 [Source:HGNC Symbol;Acc:HGNC:8043] | 5645 | 0.020 | -0.0946 | No |
| 127 | ASAP2 | "ArfGAP with SH3 domain, ankyrin repeat and PH domain 2 [Source:HGNC Symbol;Acc:HGNC:2721]" | 5742 | 0.012 | -0.1021 | No |
| 128 | HACD3 | 3-hydroxyacyl-CoA dehydratase 3 [Source:HGNC Symbol;Acc:HGNC:24175] | 5755 | 0.011 | -0.1030 | No |
| 129 | ARHGAP4 | Rho GTPase activating protein 4 [Source:HGNC Symbol;Acc:HGNC:674] | 6002 | -0.004 | -0.1225 | No |
| 130 | PREB | prolactin regulatory element binding [Source:HGNC Symbol;Acc:HGNC:9356] | 6044 | -0.008 | -0.1257 | No |
| 131 | BNIP2 | BCL2 interacting protein 2 [Source:HGNC Symbol;Acc:HGNC:1083] | 6045 | -0.008 | -0.1257 | No |
| 132 | SYDE1 | synapse defective Rho GTPase homolog 1 [Source:HGNC Symbol;Acc:HGNC:25824] | 6066 | -0.009 | -0.1272 | No |
| 133 | ARHGAP12 | Rho GTPase activating protein 12 [Source:HGNC Symbol;Acc:HGNC:16348] | 6099 | -0.013 | -0.1296 | No |
| 134 | SIPA1L1 | signal induced proliferation associated 1 like 1 [Source:HGNC Symbol;Acc:HGNC:20284] | 6151 | -0.016 | -0.1335 | No |
| 135 | TBC1D30 | TBC1 domain family member 30 [Source:HGNC Symbol;Acc:HGNC:29164] | 6231 | -0.023 | -0.1396 | No |
| 136 | HPS1 | HPS1 biogenesis of lysosomal organelles complex 3 subunit 1 [Source:HGNC Symbol;Acc:HGNC:5163] | 6251 | -0.024 | -0.1409 | No |
| 137 | ARFGEF2 | ADP ribosylation factor guanine nucleotide exchange factor 2 [Source:HGNC Symbol;Acc:HGNC:15853] | 6333 | -0.031 | -0.1471 | No |
| 138 | RASAL1 | RAS protein activator like 1 [Source:HGNC Symbol;Acc:HGNC:9873] | 6337 | -0.031 | -0.1470 | No |
| 139 | CYTH4 | cytohesin 4 [Source:HGNC Symbol;Acc:HGNC:9505] | 6494 | -0.042 | -0.1591 | No |
| 140 | GDI2 | GDP dissociation inhibitor 2 [Source:HGNC Symbol;Acc:HGNC:4227] | 6525 | -0.044 | -0.1611 | No |
| 141 | LLGL1 | LLGL scribble cell polarity complex component 1 [Source:HGNC Symbol;Acc:HGNC:6628] | 6528 | -0.044 | -0.1609 | No |
| 142 | RIN2 | Ras and Rab interactor 2 [Source:HGNC Symbol;Acc:HGNC:18750] | 6538 | -0.045 | -0.1612 | No |
| 143 | CHN1 | chimerin 1 [Source:HGNC Symbol;Acc:HGNC:1943] | 6563 | -0.047 | -0.1627 | No |
| 144 | RAP1A | "RAP1A, member of RAS oncogene family [Source:HGNC Symbol;Acc:HGNC:9855]" | 6580 | -0.048 | -0.1635 | No |
| 145 | ARHGEF12 | Rho guanine nucleotide exchange factor 12 [Source:HGNC Symbol;Acc:HGNC:14193] | 6587 | -0.049 | -0.1636 | No |
| 146 | PREX2 | "phosphatidylinositol-3,4,5-trisphosphate dependent Rac exchange factor 2 [Source:HGNC Symbol;Acc:HGNC:22950]" | 6632 | -0.052 | -0.1666 | No |
| 147 | TBXA2R | thromboxane A2 receptor [Source:HGNC Symbol;Acc:HGNC:11608] | 6651 | -0.053 | -0.1675 | No |
| 148 | ARHGAP22 | Rho GTPase activating protein 22 [Source:HGNC Symbol;Acc:HGNC:30320] | 6657 | -0.053 | -0.1675 | No |
| 149 | ARHGDIA | Rho GDP dissociation inhibitor alpha [Source:HGNC Symbol;Acc:HGNC:678] | 6664 | -0.053 | -0.1675 | No |
| 150 | WAS | WASP actin nucleation promoting factor [Source:HGNC Symbol;Acc:HGNC:12731] | 6666 | -0.054 | -0.1671 | No |
| 151 | ARHGEF28 | Rho guanine nucleotide exchange factor 28 [Source:HGNC Symbol;Acc:HGNC:30322] | 6669 | -0.054 | -0.1667 | No |
| 152 | RGS6 | regulator of G protein signaling 6 [Source:HGNC Symbol;Acc:HGNC:10002] | 6706 | -0.056 | -0.1691 | No |
| 153 | RIN1 | Ras and Rab interactor 1 [Source:HGNC Symbol;Acc:HGNC:18749] | 6730 | -0.058 | -0.1704 | No |
| 154 | HERC2 | HECT and RLD domain containing E3 ubiquitin protein ligase 2 [Source:HGNC Symbol;Acc:HGNC:4868] | 6746 | -0.059 | -0.1711 | No |
| 155 | ANKRD27 | ankyrin repeat domain 27 [Source:HGNC Symbol;Acc:HGNC:25310] | 6770 | -0.061 | -0.1724 | No |
| 156 | DENND4C | DENN domain containing 4C [Source:HGNC Symbol;Acc:HGNC:26079] | 6875 | -0.069 | -0.1800 | No |
| 157 | ARF4 | ADP ribosylation factor 4 [Source:HGNC Symbol;Acc:HGNC:655] | 6894 | -0.072 | -0.1808 | No |
| 158 | EEF1D | eukaryotic translation elongation factor 1 delta [Source:HGNC Symbol;Acc:HGNC:3211] | 6923 | -0.074 | -0.1824 | No |
| 159 | EIF5 | eukaryotic translation initiation factor 5 [Source:HGNC Symbol;Acc:HGNC:3299] | 6934 | -0.074 | -0.1825 | No |
| 160 | TSC2 | TSC complex subunit 2 [Source:HGNC Symbol;Acc:HGNC:12363] | 6946 | -0.075 | -0.1827 | No |
| 161 | IPO5 | importin 5 [Source:HGNC Symbol;Acc:HGNC:6402] | 6949 | -0.076 | -0.1822 | No |
| 162 | CYSLTR2 | cysteinyl leukotriene receptor 2 [Source:HGNC Symbol;Acc:HGNC:18274] | 6965 | -0.077 | -0.1827 | No |
| 163 | ARAP2 | "ArfGAP with RhoGAP domain, ankyrin repeat and PH domain 2 [Source:HGNC Symbol;Acc:HGNC:16924]" | 6983 | -0.079 | -0.1834 | No |
| 164 | DLC1 | DLC1 Rho GTPase activating protein [Source:HGNC Symbol;Acc:HGNC:2897] | 6984 | -0.079 | -0.1826 | No |
| 165 | ARHGAP28 | Rho GTPase activating protein 28 [Source:HGNC Symbol;Acc:HGNC:25509] | 7005 | -0.080 | -0.1835 | No |
| 166 | RANBP3 | RAN binding protein 3 [Source:HGNC Symbol;Acc:HGNC:9850] | 7043 | -0.083 | -0.1857 | No |
| 167 | AGAP1 | "ArfGAP with GTPase domain, ankyrin repeat and PH domain 1 [Source:HGNC Symbol;Acc:HGNC:16922]" | 7081 | -0.086 | -0.1879 | No |
| 168 | EPS8L1 | EPS8 like 1 [Source:HGNC Symbol;Acc:HGNC:21295] | 7083 | -0.086 | -0.1872 | No |
| 169 | SRGAP3 | SLIT-ROBO Rho GTPase activating protein 3 [Source:HGNC Symbol;Acc:HGNC:19744] | 7091 | -0.087 | -0.1870 | No |
| 170 | RIN3 | Ras and Rab interactor 3 [Source:HGNC Symbol;Acc:HGNC:18751] | 7110 | -0.089 | -0.1876 | No |
| 171 | GPSM2 | G protein signaling modulator 2 [Source:HGNC Symbol;Acc:HGNC:29501] | 7178 | -0.094 | -0.1921 | No |
| 172 | EEF1B2 | eukaryotic translation elongation factor 1 beta 2 [Source:HGNC Symbol;Acc:HGNC:3208] | 7179 | -0.095 | -0.1913 | No |
| 173 | RHOD | ras homolog family member D [Source:HGNC Symbol;Acc:HGNC:670] | 7202 | -0.096 | -0.1921 | No |
| 174 | GNB5 | G protein subunit beta 5 [Source:HGNC Symbol;Acc:HGNC:4401] | 7223 | -0.097 | -0.1929 | No |
| 175 | MTSS2 | MTSS I-BAR domain containing 2 [Source:HGNC Symbol;Acc:HGNC:25094] | 7240 | -0.098 | -0.1933 | No |
| 176 | RGL1 | ral guanine nucleotide dissociation stimulator like 1 [Source:HGNC Symbol;Acc:HGNC:30281] | 7245 | -0.099 | -0.1927 | No |
| 177 | TBC1D13 | TBC1 domain family member 13 [Source:HGNC Symbol;Acc:HGNC:25571] | 7261 | -0.100 | -0.1930 | No |
| 178 | TBC1D9B | TBC1 domain family member 9B [Source:HGNC Symbol;Acc:HGNC:29097] | 7263 | -0.100 | -0.1922 | No |
| 179 | RACGAP1 | Rac GTPase activating protein 1 [Source:HGNC Symbol;Acc:HGNC:9804] | 7269 | -0.100 | -0.1917 | No |
| 180 | OCRL | OCRL inositol polyphosphate-5-phosphatase [Source:HGNC Symbol;Acc:HGNC:8108] | 7319 | -0.103 | -0.1946 | No |
| 181 | ARHGAP5 | Rho GTPase activating protein 5 [Source:HGNC Symbol;Acc:HGNC:675] | 7357 | -0.106 | -0.1966 | No |
| 182 | DNMBP | dynamin binding protein [Source:HGNC Symbol;Acc:HGNC:30373] | 7382 | -0.109 | -0.1976 | No |
| 183 | TBC1D2 | TBC1 domain family member 2 [Source:HGNC Symbol;Acc:HGNC:18026] | 7399 | -0.111 | -0.1978 | No |
| 184 | RGL2 | ral guanine nucleotide dissociation stimulator like 2 [Source:HGNC Symbol;Acc:HGNC:9769] | 7434 | -0.114 | -0.1995 | No |
| 185 | GNAQ | G protein subunit alpha q [Source:HGNC Symbol;Acc:HGNC:4390] | 7518 | -0.120 | -0.2051 | No |
| 186 | EVI5 | ecotropic viral integration site 5 [Source:HGNC Symbol;Acc:HGNC:3501] | 7616 | -0.128 | -0.2116 | No |
| 187 | EIF2B1 | eukaryotic translation initiation factor 2B subunit alpha [Source:HGNC Symbol;Acc:HGNC:3257] | 7617 | -0.128 | -0.2105 | No |
| 188 | RGS14 | regulator of G protein signaling 14 [Source:HGNC Symbol;Acc:HGNC:9996] | 7719 | -0.135 | -0.2173 | No |
| 189 | AGAP2 | "ArfGAP with GTPase domain, ankyrin repeat and PH domain 2 [Source:HGNC Symbol;Acc:HGNC:16921]" | 7732 | -0.136 | -0.2170 | No |
| 190 | AGFG2 | ArfGAP with FG repeats 2 [Source:HGNC Symbol;Acc:HGNC:5177] | 7796 | -0.141 | -0.2208 | No |
| 191 | CHN2 | chimerin 2 [Source:HGNC Symbol;Acc:HGNC:1944] | 7847 | -0.144 | -0.2235 | No |
| 192 | HPS4 | HPS4 biogenesis of lysosomal organelles complex 3 subunit 2 [Source:HGNC Symbol;Acc:HGNC:15844] | 7868 | -0.146 | -0.2238 | No |
| 193 | ARHGEF6 | Rac/Cdc42 guanine nucleotide exchange factor 6 [Source:HGNC Symbol;Acc:HGNC:685] | 7900 | -0.147 | -0.2249 | No |
| 194 | RAP1GAP | RAP1 GTPase activating protein [Source:HGNC Symbol;Acc:HGNC:9858] | 7910 | -0.148 | -0.2243 | No |
| 195 | RAP1GAP2 | RAP1 GTPase activating protein 2 [Source:HGNC Symbol;Acc:HGNC:29176] | 7955 | -0.152 | -0.2264 | No |
| 196 | RAB3GAP2 | RAB3 GTPase activating non-catalytic protein subunit 2 [Source:HGNC Symbol;Acc:HGNC:17168] | 7957 | -0.152 | -0.2252 | No |
| 197 | RALGPS2 | Ral GEF with PH domain and SH3 binding motif 2 [Source:HGNC Symbol;Acc:HGNC:30279] | 7987 | -0.154 | -0.2261 | No |
| 198 | GPSM3 | G protein signaling modulator 3 [Source:HGNC Symbol;Acc:HGNC:13945] | 8045 | -0.159 | -0.2292 | No |
| 199 | SOS2 | SOS Ras/Rho guanine nucleotide exchange factor 2 [Source:HGNC Symbol;Acc:HGNC:11188] | 8058 | -0.160 | -0.2287 | No |
| 200 | SRGAP2 | SLIT-ROBO Rho GTPase activating protein 2 [Source:HGNC Symbol;Acc:HGNC:19751] | 8116 | -0.164 | -0.2318 | No |
| 201 | RABEP1 | "rabaptin, RAB GTPase binding effector protein 1 [Source:HGNC Symbol;Acc:HGNC:17677]" | 8165 | -0.168 | -0.2341 | No |
| 202 | EIF2B5 | eukaryotic translation initiation factor 2B subunit epsilon [Source:HGNC Symbol;Acc:HGNC:3261] | 8168 | -0.168 | -0.2327 | No |
| 203 | ABR | ABR activator of RhoGEF and GTPase [Source:HGNC Symbol;Acc:HGNC:81] | 8184 | -0.170 | -0.2324 | No |
| 204 | TBC1D15 | TBC1 domain family member 15 [Source:HGNC Symbol;Acc:HGNC:25694] | 8197 | -0.171 | -0.2318 | No |
| 205 | ADRB1 | adrenoceptor beta 1 [Source:HGNC Symbol;Acc:HGNC:285] | 8212 | -0.172 | -0.2314 | No |
| 206 | TBC1D17 | TBC1 domain family member 17 [Source:HGNC Symbol;Acc:HGNC:25699] | 8230 | -0.173 | -0.2312 | No |
| 207 | STXBP5L | syntaxin binding protein 5L [Source:HGNC Symbol;Acc:HGNC:30757] | 8240 | -0.174 | -0.2303 | No |
| 208 | IQGAP1 | IQ motif containing GTPase activating protein 1 [Source:HGNC Symbol;Acc:HGNC:6110] | 8276 | -0.177 | -0.2315 | No |
| 209 | DENND4B | DENN domain containing 4B [Source:HGNC Symbol;Acc:HGNC:29044] | 8315 | -0.179 | -0.2330 | No |
| 210 | ARHGAP17 | Rho GTPase activating protein 17 [Source:HGNC Symbol;Acc:HGNC:18239] | 8325 | -0.180 | -0.2321 | No |
| 211 | DEPDC5 | "DEP domain containing 5, GATOR1 subcomplex subunit [Source:HGNC Symbol;Acc:HGNC:18423]" | 8331 | -0.180 | -0.2308 | No |
| 212 | RASGRP1 | RAS guanyl releasing protein 1 [Source:HGNC Symbol;Acc:HGNC:9878] | 8352 | -0.182 | -0.2308 | No |
| 213 | ARHGAP32 | Rho GTPase activating protein 32 [Source:HGNC Symbol;Acc:HGNC:17399] | 8370 | -0.183 | -0.2305 | No |
| 214 | DOCK5 | dedicator of cytokinesis 5 [Source:HGNC Symbol;Acc:HGNC:23476] | 8380 | -0.184 | -0.2296 | No |
| 215 | RHOF | "ras homolog family member F, filopodia associated [Source:HGNC Symbol;Acc:HGNC:15703]" | 8403 | -0.186 | -0.2296 | No |
| 216 | SLIT2 | slit guidance ligand 2 [Source:HGNC Symbol;Acc:HGNC:11086] | 8449 | -0.190 | -0.2315 | No |
| 217 | GBF1 | golgi brefeldin A resistant guanine nucleotide exchange factor 1 [Source:HGNC Symbol;Acc:HGNC:4181] | 8461 | -0.191 | -0.2307 | No |
| 218 | DNM1L | dynamin 1 like [Source:HGNC Symbol;Acc:HGNC:2973] | 8499 | -0.194 | -0.2319 | No |
| 219 | TBC1D22B | TBC1 domain family member 22B [Source:HGNC Symbol;Acc:HGNC:21602] | 8571 | -0.199 | -0.2358 | No |
| 220 | IQGAP2 | IQ motif containing GTPase activating protein 2 [Source:HGNC Symbol;Acc:HGNC:6111] | 8598 | -0.201 | -0.2360 | No |
| 221 | RAPGEF5 | Rap guanine nucleotide exchange factor 5 [Source:HGNC Symbol;Acc:HGNC:16862] | 8622 | -0.202 | -0.2360 | No |
| 222 | ECT2 | epithelial cell transforming 2 [Source:HGNC Symbol;Acc:HGNC:3155] | 8647 | -0.204 | -0.2361 | No |
| 223 | FGD2 | "FYVE, RhoGEF and PH domain containing 2 [Source:HGNC Symbol;Acc:HGNC:3664]" | 8696 | -0.209 | -0.2381 | No |
| 224 | ARHGEF7 | Rho guanine nucleotide exchange factor 7 [Source:HGNC Symbol;Acc:HGNC:15607] | 8746 | -0.212 | -0.2401 | No |
| 225 | DENND2A | DENN domain containing 2A [Source:HGNC Symbol;Acc:HGNC:22212] | 8776 | -0.215 | -0.2404 | No |
| 226 | ARHGAP35 | Rho GTPase activating protein 35 [Source:HGNC Symbol;Acc:HGNC:4591] | 8835 | -0.220 | -0.2431 | No |
| 227 | FLCN | folliculin [Source:HGNC Symbol;Acc:HGNC:27310] | 8848 | -0.221 | -0.2420 | No |
| 228 | ITSN2 | intersectin 2 [Source:HGNC Symbol;Acc:HGNC:6184] | 8922 | -0.226 | -0.2458 | No |
| 229 | TNK2 | tyrosine kinase non receptor 2 [Source:HGNC Symbol;Acc:HGNC:19297] | 8955 | -0.228 | -0.2463 | No |
| 230 | RASGRF1 | Ras protein specific guanine nucleotide releasing factor 1 [Source:HGNC Symbol;Acc:HGNC:9875] | 8982 | -0.230 | -0.2463 | No |
| 231 | RAPGEF6 | Rap guanine nucleotide exchange factor 6 [Source:HGNC Symbol;Acc:HGNC:20655] | 8993 | -0.231 | -0.2450 | No |
| 232 | DENND4A | DENN domain containing 4A [Source:HGNC Symbol;Acc:HGNC:24321] | 8999 | -0.231 | -0.2434 | No |
| 233 | ARHGEF10L | Rho guanine nucleotide exchange factor 10 like [Source:HGNC Symbol;Acc:HGNC:25540] | 9020 | -0.233 | -0.2429 | No |
| 234 | BCAR3 | "BCAR3 adaptor protein, NSP family member [Source:HGNC Symbol;Acc:HGNC:973]" | 9139 | -0.243 | -0.2501 | No |
| 235 | RASA2 | RAS p21 protein activator 2 [Source:HGNC Symbol;Acc:HGNC:9872] | 9153 | -0.244 | -0.2489 | No |
| 236 | RASAL2 | RAS protein activator like 2 [Source:HGNC Symbol;Acc:HGNC:9874] | 9161 | -0.245 | -0.2473 | No |
| 237 | DNAJA3 | DnaJ heat shock protein family (Hsp40) member A3 [Source:HGNC Symbol;Acc:HGNC:11808] | 9172 | -0.246 | -0.2459 | No |
| 238 | NF1 | neurofibromin 1 [Source:HGNC Symbol;Acc:HGNC:7765] | 9194 | -0.247 | -0.2453 | No |
| 239 | GIT1 | GIT ArfGAP 1 [Source:HGNC Symbol;Acc:HGNC:4272] | 9236 | -0.251 | -0.2463 | No |
| 240 | RIC8B | RIC8 guanine nucleotide exchange factor B [Source:HGNC Symbol;Acc:HGNC:25555] | 9306 | -0.256 | -0.2495 | No |
| 241 | ARHGAP1 | Rho GTPase activating protein 1 [Source:HGNC Symbol;Acc:HGNC:673] | 9307 | -0.256 | -0.2472 | No |
| 242 | GPS2 | G protein pathway suppressor 2 [Source:HGNC Symbol;Acc:HGNC:4550] | 9338 | -0.258 | -0.2473 | No |
| 243 | RGS5 | regulator of G protein signaling 5 [Source:HGNC Symbol;Acc:HGNC:10001] | 9432 | -0.267 | -0.2523 | No |
| 244 | DOCK9 | dedicator of cytokinesis 9 [Source:HGNC Symbol;Acc:HGNC:14132] | 9545 | -0.278 | -0.2587 | No |
| 245 | GIT2 | GIT ArfGAP 2 [Source:HGNC Symbol;Acc:HGNC:4273] | 9552 | -0.278 | -0.2567 | No |
| 246 | RASGRP3 | RAS guanyl releasing protein 3 [Source:HGNC Symbol;Acc:HGNC:14545] | 9603 | -0.284 | -0.2581 | No |
| 247 | KALRN | kalirin RhoGEF kinase [Source:HGNC Symbol;Acc:HGNC:4814] | 9649 | -0.287 | -0.2591 | No |
| 248 | DENND5A | DENN domain containing 5A [Source:HGNC Symbol;Acc:HGNC:19344] | 9714 | -0.293 | -0.2616 | No |
| 249 | BCR | BCR activator of RhoGEF and GTPase [Source:HGNC Symbol;Acc:HGNC:1014] | 9830 | -0.304 | -0.2680 | No |
| 250 | TBC1D10B | TBC1 domain family member 10B [Source:HGNC Symbol;Acc:HGNC:24510] | 9880 | -0.310 | -0.2692 | Yes |
| 251 | ARHGAP29 | Rho GTPase activating protein 29 [Source:HGNC Symbol;Acc:HGNC:30207] | 9916 | -0.313 | -0.2691 | Yes |
| 252 | RABGAP1 | RAB GTPase activating protein 1 [Source:HGNC Symbol;Acc:HGNC:17155] | 9928 | -0.315 | -0.2672 | Yes |
| 253 | GRTP1 | growth hormone regulated TBC protein 1 [Source:HGNC Symbol;Acc:HGNC:20310] | 9930 | -0.315 | -0.2644 | Yes |
| 254 | ARHGEF26 | Rho guanine nucleotide exchange factor 26 [Source:HGNC Symbol;Acc:HGNC:24490] | 9979 | -0.319 | -0.2654 | Yes |
| 255 | SH3BP1 | SH3 domain binding protein 1 [Source:HGNC Symbol;Acc:HGNC:10824] | 10016 | -0.323 | -0.2653 | Yes |
| 256 | SWAP70 | switching B cell complex subunit SWAP70 [Source:HGNC Symbol;Acc:HGNC:17070] | 10019 | -0.323 | -0.2626 | Yes |
| 257 | ARHGEF11 | Rho guanine nucleotide exchange factor 11 [Source:HGNC Symbol;Acc:HGNC:14580] | 10056 | -0.326 | -0.2625 | Yes |
| 258 | RGS12 | regulator of G protein signaling 12 [Source:HGNC Symbol;Acc:HGNC:9994] | 10163 | -0.337 | -0.2680 | Yes |
| 259 | STARD13 | StAR related lipid transfer domain containing 13 [Source:HGNC Symbol;Acc:HGNC:19164] | 10183 | -0.339 | -0.2664 | Yes |
| 260 | PLEKHG3 | pleckstrin homology and RhoGEF domain containing G3 [Source:HGNC Symbol;Acc:HGNC:20364] | 10217 | -0.343 | -0.2660 | Yes |
| 261 | TBCD | tubulin folding cofactor D [Source:HGNC Symbol;Acc:HGNC:11581] | 10252 | -0.346 | -0.2656 | Yes |
| 262 | ASAP1 | "ArfGAP with SH3 domain, ankyrin repeat and PH domain 1 [Source:HGNC Symbol;Acc:HGNC:2720]" | 10276 | -0.349 | -0.2643 | Yes |
| 263 | ARHGEF16 | Rho guanine nucleotide exchange factor 16 [Source:HGNC Symbol;Acc:HGNC:15515] | 10278 | -0.349 | -0.2612 | Yes |
| 264 | RALGDS | ral guanine nucleotide dissociation stimulator [Source:HGNC Symbol;Acc:HGNC:9842] | 10287 | -0.349 | -0.2587 | Yes |
| 265 | RANGAP1 | Ran GTPase activating protein 1 [Source:HGNC Symbol;Acc:HGNC:9854] | 10288 | -0.349 | -0.2556 | Yes |
| 266 | RP2 | RP2 activator of ARL3 GTPase [Source:HGNC Symbol;Acc:HGNC:10274] | 10354 | -0.356 | -0.2575 | Yes |
| 267 | TRIO | trio Rho guanine nucleotide exchange factor [Source:HGNC Symbol;Acc:HGNC:12303] | 10362 | -0.357 | -0.2549 | Yes |
| 268 | ARFGAP3 | ADP ribosylation factor GTPase activating protein 3 [Source:HGNC Symbol;Acc:HGNC:661] | 10382 | -0.359 | -0.2532 | Yes |
| 269 | SGSM2 | small G protein signaling modulator 2 [Source:HGNC Symbol;Acc:HGNC:29026] | 10403 | -0.361 | -0.2515 | Yes |
| 270 | SBF1 | SET binding factor 1 [Source:HGNC Symbol;Acc:HGNC:10542] | 10406 | -0.361 | -0.2484 | Yes |
| 271 | GAPVD1 | GTPase activating protein and VPS9 domains 1 [Source:HGNC Symbol;Acc:HGNC:23375] | 10410 | -0.362 | -0.2454 | Yes |
| 272 | RGS1 | regulator of G protein signaling 1 [Source:HGNC Symbol;Acc:HGNC:9991] | 10422 | -0.363 | -0.2430 | Yes |
| 273 | ARFGEF1 | ADP ribosylation factor guanine nucleotide exchange factor 1 [Source:HGNC Symbol;Acc:HGNC:15772] | 10477 | -0.371 | -0.2440 | Yes |
| 274 | GPS1 | G protein pathway suppressor 1 [Source:HGNC Symbol;Acc:HGNC:4549] | 10536 | -0.377 | -0.2452 | Yes |
| 275 | ARHGAP8 | Rho GTPase activating protein 8 [Source:HGNC Symbol;Acc:HGNC:677] | 10551 | -0.378 | -0.2429 | Yes |
| 276 | RABEP2 | "rabaptin, RAB GTPase binding effector protein 2 [Source:HGNC Symbol;Acc:HGNC:24817]" | 10568 | -0.380 | -0.2408 | Yes |
| 277 | IQSEC3 | IQ motif and Sec7 domain ArfGEF 3 [Source:HGNC Symbol;Acc:HGNC:29193] | 10573 | -0.381 | -0.2377 | Yes |
| 278 | RASA3 | RAS p21 protein activator 3 [Source:HGNC Symbol;Acc:HGNC:20331] | 10574 | -0.381 | -0.2343 | Yes |
| 279 | KRIT1 | KRIT1 ankyrin repeat containing [Source:HGNC Symbol;Acc:HGNC:1573] | 10575 | -0.381 | -0.2308 | Yes |
| 280 | RGPD5 | RANBP2 like and GRIP domain containing 5 [Source:HGNC Symbol;Acc:HGNC:32418] | 10604 | -0.384 | -0.2296 | Yes |
| 281 | IPO7 | importin 7 [Source:HGNC Symbol;Acc:HGNC:9852] | 10662 | -0.389 | -0.2307 | Yes |
| 282 | PLXNB1 | plexin B1 [Source:HGNC Symbol;Acc:HGNC:9103] | 10682 | -0.391 | -0.2287 | Yes |
| 283 | ARHGAP44 | Rho GTPase activating protein 44 [Source:HGNC Symbol;Acc:HGNC:29096] | 10733 | -0.397 | -0.2291 | Yes |
| 284 | MCF2L | MCF.2 cell line derived transforming sequence like [Source:HGNC Symbol;Acc:HGNC:14576] | 10755 | -0.399 | -0.2271 | Yes |
| 285 | ARHGEF2 | Rho/Rac guanine nucleotide exchange factor 2 [Source:HGNC Symbol;Acc:HGNC:682] | 10808 | -0.406 | -0.2276 | Yes |
| 286 | ARFGAP1 | ADP ribosylation factor GTPase activating protein 1 [Source:HGNC Symbol;Acc:HGNC:15852] | 10827 | -0.408 | -0.2254 | Yes |
| 287 | MYO9B | myosin IXB [Source:HGNC Symbol;Acc:HGNC:7609] | 10864 | -0.412 | -0.2246 | Yes |
| 288 | FGD1 | "FYVE, RhoGEF and PH domain containing 1 [Source:HGNC Symbol;Acc:HGNC:3663]" | 10892 | -0.416 | -0.2230 | Yes |
| 289 | SIPA1L3 | signal induced proliferation associated 1 like 3 [Source:HGNC Symbol;Acc:HGNC:23801] | 11036 | -0.435 | -0.2305 | Yes |
| 290 | RAPGEF2 | Rap guanine nucleotide exchange factor 2 [Source:HGNC Symbol;Acc:HGNC:16854] | 11041 | -0.435 | -0.2269 | Yes |
| 291 | RGS9 | regulator of G protein signaling 9 [Source:HGNC Symbol;Acc:HGNC:10004] | 11044 | -0.436 | -0.2231 | Yes |
| 292 | ARHGAP33 | Rho GTPase activating protein 33 [Source:HGNC Symbol;Acc:HGNC:23085] | 11130 | -0.447 | -0.2259 | Yes |
| 293 | RPGR | retinitis pigmentosa GTPase regulator [Source:HGNC Symbol;Acc:HGNC:10295] | 11166 | -0.453 | -0.2246 | Yes |
| 294 | ARHGAP6 | Rho GTPase activating protein 6 [Source:HGNC Symbol;Acc:HGNC:676] | 11187 | -0.456 | -0.2221 | Yes |
| 295 | ACAP2 | "ArfGAP with coiled-coil, ankyrin repeat and PH domains 2 [Source:HGNC Symbol;Acc:HGNC:16469]" | 11229 | -0.461 | -0.2212 | Yes |
| 296 | RGS17 | regulator of G protein signaling 17 [Source:HGNC Symbol;Acc:HGNC:14088] | 11249 | -0.463 | -0.2185 | Yes |
| 297 | ARHGEF17 | Rho guanine nucleotide exchange factor 17 [Source:HGNC Symbol;Acc:HGNC:21726] | 11323 | -0.474 | -0.2201 | Yes |
| 298 | ITSN1 | intersectin 1 [Source:HGNC Symbol;Acc:HGNC:6183] | 11369 | -0.482 | -0.2193 | Yes |
| 299 | MYCBP2 | MYC binding protein 2 [Source:HGNC Symbol;Acc:HGNC:23386] | 11377 | -0.483 | -0.2156 | Yes |
| 300 | RAPGEF3 | Rap guanine nucleotide exchange factor 3 [Source:HGNC Symbol;Acc:HGNC:16629] | 11380 | -0.483 | -0.2114 | Yes |
| 301 | VAV2 | vav guanine nucleotide exchange factor 2 [Source:HGNC Symbol;Acc:HGNC:12658] | 11413 | -0.489 | -0.2095 | Yes |
| 302 | ARHGEF15 | Rho guanine nucleotide exchange factor 15 [Source:HGNC Symbol;Acc:HGNC:15590] | 11489 | -0.500 | -0.2110 | Yes |
| 303 | TBC1D8 | TBC1 domain family member 8 [Source:HGNC Symbol;Acc:HGNC:17791] | 11490 | -0.500 | -0.2065 | Yes |
| 304 | IQSEC2 | IQ motif and Sec7 domain ArfGEF 2 [Source:HGNC Symbol;Acc:HGNC:29059] | 11494 | -0.500 | -0.2023 | Yes |
| 305 | VPS9D1 | VPS9 domain containing 1 [Source:HGNC Symbol;Acc:HGNC:13526] | 11495 | -0.501 | -0.1977 | Yes |
| 306 | DEF6 | DEF6 guanine nucleotide exchange factor [Source:HGNC Symbol;Acc:HGNC:2760] | 11535 | -0.505 | -0.1963 | Yes |
| 307 | SH3BP5 | SH3 domain binding protein 5 [Source:HGNC Symbol;Acc:HGNC:10827] | 11545 | -0.506 | -0.1925 | Yes |
| 308 | USP6 | ubiquitin specific peptidase 6 [Source:HGNC Symbol;Acc:HGNC:12629] | 11570 | -0.511 | -0.1898 | Yes |
| 309 | PSD | pleckstrin and Sec7 domain containing [Source:HGNC Symbol;Acc:HGNC:9507] | 11597 | -0.515 | -0.1872 | Yes |
| 310 | DENND5B | DENN domain containing 5B [Source:HGNC Symbol;Acc:HGNC:28338] | 11631 | -0.522 | -0.1852 | Yes |
| 311 | RALGAPA1 | Ral GTPase activating protein catalytic subunit alpha 1 [Source:HGNC Symbol;Acc:HGNC:17770] | 11779 | -0.546 | -0.1920 | Yes |
| 312 | CHM | CHM Rab escort protein [Source:HGNC Symbol;Acc:HGNC:1940] | 11798 | -0.549 | -0.1885 | Yes |
| 313 | GDI1 | GDP dissociation inhibitor 1 [Source:HGNC Symbol;Acc:HGNC:4226] | 11803 | -0.550 | -0.1838 | Yes |
| 314 | CYTH1 | cytohesin 1 [Source:HGNC Symbol;Acc:HGNC:9501] | 11804 | -0.550 | -0.1789 | Yes |
| 315 | ARHGAP19 | Rho GTPase activating protein 19 [Source:HGNC Symbol;Acc:HGNC:23724] | 11822 | -0.553 | -0.1753 | Yes |
| 316 | RAPGEF4 | Rap guanine nucleotide exchange factor 4 [Source:HGNC Symbol;Acc:HGNC:16626] | 11963 | -0.586 | -0.1812 | Yes |
| 317 | ARHGAP10 | Rho GTPase activating protein 10 [Source:HGNC Symbol;Acc:HGNC:26099] | 11998 | -0.592 | -0.1785 | Yes |
| 318 | CDC42SE1 | CDC42 small effector 1 [Source:HGNC Symbol;Acc:HGNC:17719] | 12018 | -0.596 | -0.1747 | Yes |
| 319 | TBC1D3F | TBC1 domain family member 3F [Source:HGNC Symbol;Acc:HGNC:18257] | 12019 | -0.597 | -0.1693 | Yes |
| 320 | AGFG1 | ArfGAP with FG repeats 1 [Source:HGNC Symbol;Acc:HGNC:5175] | 12035 | -0.602 | -0.1651 | Yes |
| 321 | RGS2 | regulator of G protein signaling 2 [Source:HGNC Symbol;Acc:HGNC:9998] | 12039 | -0.603 | -0.1599 | Yes |
| 322 | SOS1 | SOS Ras/Rac guanine nucleotide exchange factor 1 [Source:HGNC Symbol;Acc:HGNC:11187] | 12041 | -0.603 | -0.1546 | Yes |
| 323 | MADD | MAP kinase activating death domain [Source:HGNC Symbol;Acc:HGNC:6766] | 12056 | -0.609 | -0.1502 | Yes |
| 324 | ARHGEF38 | Rho guanine nucleotide exchange factor 38 [Source:HGNC Symbol;Acc:HGNC:25968] | 12091 | -0.618 | -0.1474 | Yes |
| 325 | EPS8L3 | EPS8 like 3 [Source:HGNC Symbol;Acc:HGNC:21297] | 12097 | -0.619 | -0.1422 | Yes |
| 326 | RASA4 | RAS p21 protein activator 4 [Source:HGNC Symbol;Acc:HGNC:23181] | 12099 | -0.619 | -0.1367 | Yes |
| 327 | RASGRP2 | RAS guanyl releasing protein 2 [Source:HGNC Symbol;Acc:HGNC:9879] | 12124 | -0.625 | -0.1330 | Yes |
| 328 | CHML | CHM like Rab escort protein [Source:HGNC Symbol;Acc:HGNC:1941] | 12132 | -0.626 | -0.1279 | Yes |
| 329 | MYO9A | myosin IXA [Source:HGNC Symbol;Acc:HGNC:7608] | 12166 | -0.635 | -0.1248 | Yes |
| 330 | WASL | WASP like actin nucleation promoting factor [Source:HGNC Symbol;Acc:HGNC:12735] | 12207 | -0.647 | -0.1222 | Yes |
| 331 | RASA4B | RAS p21 protein activator 4B [Source:HGNC Symbol;Acc:HGNC:35202] | 12265 | -0.667 | -0.1207 | Yes |
| 332 | ADPRH | ADP-ribosylarginine hydrolase [Source:HGNC Symbol;Acc:HGNC:269] | 12277 | -0.670 | -0.1156 | Yes |
| 333 | RGP1 | "RGP1 homolog, RAB6A GEF complex partner 1 [Source:HGNC Symbol;Acc:HGNC:21965]" | 12302 | -0.677 | -0.1114 | Yes |
| 334 | FARP2 | "FERM, ARH/RhoGEF and pleckstrin domain protein 2 [Source:HGNC Symbol;Acc:HGNC:16460]" | 12318 | -0.682 | -0.1065 | Yes |
| 335 | ARAP3 | "ArfGAP with RhoGAP domain, ankyrin repeat and PH domain 3 [Source:HGNC Symbol;Acc:HGNC:24097]" | 12386 | -0.704 | -0.1055 | Yes |
| 336 | LARS1 | leucyl-tRNA synthetase 1 [Source:HGNC Symbol;Acc:HGNC:6512] | 12415 | -0.712 | -0.1013 | Yes |
| 337 | ARHGAP26 | Rho GTPase activating protein 26 [Source:HGNC Symbol;Acc:HGNC:17073] | 12438 | -0.720 | -0.0966 | Yes |
| 338 | DENND3 | DENN domain containing 3 [Source:HGNC Symbol;Acc:HGNC:29134] | 12475 | -0.739 | -0.0928 | Yes |
| 339 | SGSM3 | small G protein signaling modulator 3 [Source:HGNC Symbol;Acc:HGNC:25228] | 12515 | -0.752 | -0.0892 | Yes |
| 340 | RGS16 | regulator of G protein signaling 16 [Source:HGNC Symbol;Acc:HGNC:9997] | 12523 | -0.759 | -0.0829 | Yes |
| 341 | NPRL3 | "NPR3 like, GATOR1 complex subunit [Source:HGNC Symbol;Acc:HGNC:14124]" | 12570 | -0.781 | -0.0795 | Yes |
| 342 | AKAP13 | A-kinase anchoring protein 13 [Source:HGNC Symbol;Acc:HGNC:371] | 12572 | -0.782 | -0.0726 | Yes |
| 343 | AGAP4 | "ArfGAP with GTPase domain, ankyrin repeat and PH domain 4 [Source:HGNC Symbol;Acc:HGNC:23459]" | 12608 | -0.803 | -0.0681 | Yes |
| 344 | RANBP2 | RAN binding protein 2 [Source:HGNC Symbol;Acc:HGNC:9848] | 12609 | -0.804 | -0.0609 | Yes |
| 345 | CYTH3 | cytohesin 3 [Source:HGNC Symbol;Acc:HGNC:9504] | 12632 | -0.814 | -0.0553 | Yes |
| 346 | DOCK6 | dedicator of cytokinesis 6 [Source:HGNC Symbol;Acc:HGNC:19189] | 12633 | -0.814 | -0.0480 | Yes |
| 347 | ARHGEF1 | Rho guanine nucleotide exchange factor 1 [Source:HGNC Symbol;Acc:HGNC:681] | 12673 | -0.846 | -0.0435 | Yes |
| 348 | RCC1 | regulator of chromosome condensation 1 [Source:HGNC Symbol;Acc:HGNC:1913] | 12699 | -0.864 | -0.0378 | Yes |
| 349 | RIMS1 | regulating synaptic membrane exocytosis 1 [Source:HGNC Symbol;Acc:HGNC:17282] | 12704 | -0.866 | -0.0303 | Yes |
| 350 | CDC42EP2 | CDC42 effector protein 2 [Source:HGNC Symbol;Acc:HGNC:16263] | 12756 | -0.920 | -0.0261 | Yes |
| 351 | SH2D3A | SH2 domain containing 3A [Source:HGNC Symbol;Acc:HGNC:16885] | 12784 | -0.943 | -0.0197 | Yes |
| 352 | JUN | "Jun proto-oncogene, AP-1 transcription factor subunit [Source:HGNC Symbol;Acc:HGNC:6204]" | 12813 | -0.994 | -0.0130 | Yes |
| 353 | RAPGEF1 | Rap guanine nucleotide exchange factor 1 [Source:HGNC Symbol;Acc:HGNC:4568] | 12860 | -1.098 | -0.0068 | Yes |
| 354 | GNA13 | G protein subunit alpha 13 [Source:HGNC Symbol;Acc:HGNC:4381] | 12862 | -1.103 | 0.0030 | Yes |
Table: GSEA details [plain text format]

  

Fig 2: GOMF\_NUCLEOSIDE\_TRIPHOSPHATASE\_REGULATOR\_ACTIVITY      
 Blue-Pink O' Gram in the Space of the Analyzed GeneSet

  

Fig 3: GOMF\_NUCLEOSIDE\_TRIPHOSPHATASE\_REGULATOR\_ACTIVITY: Random ES distribution      
 Gene set null distribution of ES for **GOMF\_NUCLEOSIDE\_TRIPHOSPHATASE\_REGULATOR\_ACTIVITY**
